# Exposure to Political Disparagement Humor and Its Impact on Trust in Politicians: How Long Does It Last?

**DOI:** 10.3389/fpsyg.2017.02236

**Published:** 2017-12-22

**Authors:** Andrés Mendiburo-Seguel, Salvador Vargas, Andrés Rubio

**Affiliations:** ^1^Facultad de Educación, Universidad Andrés Bello, Santiago, Chile; ^2^Department of Psychology, University of Girona, Girona, Spain; ^3^Facultad de Psicología, Universidad Diego Portales, Santiago, Chile; ^4^Fundación Centro de Estudios Cuantitativos, Santiago, Chile

**Keywords:** political humor, trust in politicians, disparagement humor, elaboration likelihood model, disposition theory

## Abstract

The experimental research that looks into the effects of political humor on an individual’s attitudes toward politics and politicians does not evaluate its long-term effects. With this in mind, this study aims to determine the possible effects that being exposed to humor which belittles politicians may have on an ordinary citizen’s trust in them, while at the same time it observes the possible effects that such exposure has on them and the time such effects last. Two hypotheses were tested. The first one was that humor involves less cognitive elaboration, which leads to a short-term impact on the perception of the individual. The second one was that the repetition of a message can augment the swing of such message. Also, a series of elements regarding disposition toward politicians and political affiliation were considered. Two experiments were designed. The first experiment, (*N* = 94), considered three groups: one exposed to political disparagement humor; one control group exposed to disparagement humor against non-politician subjects; and a control group exposed to a non-humorous political video. Trust in politicians was evaluated first at baseline, then immediately after the experimental manipulation, and once again a week after the experimental manipulation had happened. In the second experiment (*N* = 146), participants were randomly assigned to one experimental and two control groups. The trust in politicians of the three groups was estimated and they were sent political cartoons, non-political cartoons, and newspaper headlines regarding political topics twice a day for a week via WhatsApp. Trust in politicians among the three groups was assessed again after 1 week, and for a third time 1 week after that. As a result, it was observed that a one-off exposure to political disparagement humor affects trust in politicians negatively; however, the effect it attains is short-lived and can be explained through the political content of the item and not only humor. Also, being exposed to cartoons constantly for a week had no impact whatsoever on the way politics and politicians were perceived during the time the experiment was carried out. Possible explanations for these findings are discussed.

## Introduction

The main aim of this research is to observe the possible effects that being exposed to political disparagement humor has on trust in politicians. This has been studied (e.g., Olson et al., 1999; Baumgartner et al., 2012), but the possible duration of effects has not been considered before, which provided the focus of the research reported here. Most of the empirical research on the topic of humor and politics has considered short-term effects when using experimental designs (e.g., Hobden and Olson, 1994; Olson et al., 1999; Holbert et al., 2007; Baumgartner and Morris, 2008; Kim and Vishak, 2008; Xenos and Becker, 2009; Becker, 2011, 2014; Becker and Haller, 2014) or non-experimental designs (e.g., Moy et al., 2005, 2006; Kenski and Stroud, 2006; Cao, 2008; Baumgartner, 2013) but the duration of the possible effects has not been studied using follow-up assessments after the initial post-exposure assessments. In this view, the two experiments presented here seek not only to assess the effect of political disparagement humor but also to observe its possible consequences 1 week after the exposure had occurred.

## Disparagement Humor

Disparagement humor (Zillmann, 1983) refers to the use of humor to denigrate a given target (Ferguson and Ford, 2008; for a review, see Wicker et al., 1980). According to Ferguson and Ford (2008, p. 283), “disparagement humor refers to remarks that (are intended to) elicit amusement through the denigration, derogation, or belittlement of a given target (e.g., individuals, social groups, political ideologies, material possessions),” enabling the expression and satisfaction of aggressive impulses in a socially acceptable way (Ferguson and Ford, 2008).

Disparagement humor is strongly related to prejudice (Ford and Ferguson, 2004), given that humor communication is not intended to be evaluated in a serious way. When a targeted group is disparaged, people will be less likely to be critical of the content of that message and will, consequently, adopt the attitudes implicit in the message (Nabi et al., 2007). This, in turn, could lead to a lower threshold to accept discrimination. This principle can be extended to the political arena, considering that when denigration is expressed in a humorous manner people will be in a good disposition to accept a negative description of politicians.

Although the use of this type of humor can be interpreted within the framework of either psychoanalytic or superiority theories, it has also been analyzed within social identity theory (Tajfel, 1974, 1979, 1982; Tajfel and Billig, 1974). According to this view, people construct their social identity through the comparison of the groups they belong to (in-groups) with other groups (out-groups). This comparison serves to achieve a positive distinctiveness, enhancing features that favor the in-group over the out-group arbitrarily. In this context, disparagement humor may be used as a way to obtain a positive distinctiveness, especially in the face of perceived identity threats from the out-groups, considering that people should be more amused when disparagement targets an out-group, as suggested in the literature (Wolff et al., 1934).

Another way of understanding the joy that disparagement humor causes can be found in Zillmann and Cantor’s (1976) disposition theory of humor and disposition theory of mirth. Disposition theory of humor is a conceptual framework deriving from disparagement humor (Wicker et al., 1980), but it relates better to superiority theories than to social identity theories. According to disposition theory, the response to humorous stimuli depends on the affective disposition toward the targeted person or group (McGhee and Lloyd, 1981; Becker, 2014). This theory posits that people react affectively to any target in a continuum that ranges from extreme positivity to extreme negativity, through a neutral point. In that context, it is considered that the closer the targeted group is toward the negative pole, the more amusement, humor, or mirth will be perceived by the individuals (Zillmann and Cantor, 1976).

The literature suggests that humor in general, and disparagement humor in particular, can be enjoyed because it acts as a kind of “mental balm,” which allows the sender to deliver information by bringing about “high spirits,” thus creating greater possibilities for the messages to be received effectively (Sternthal and Craig, 1973; Kuiper et al., 1995). This would generate positive affect that would inhibit counterargument (Mackie and Worth, 1989), which can also be understood through the elaboration likelihood model.

The elaboration likelihood model posits that individuals are not always either thoughtful or mindless about messages (Cacioppo and Petty, 1984; Petty and Cacioppo, 1984a, 1986; Cacioppo et al., 1985). Instead, different factors influence the way in which people process the information they obtain from the environment. When these factors or sources enhance interest in the received message, the elaboration likelihood is higher, so people will be more likely to process and think carefully about the arguments proposed by the message. Conversely, when interest is lowered, the elaboration likelihood is also lower, which will lead to the opposite consequence. Therefore, messages are processed in two ways: a central route, where the message is as persuasive as the argument is adequate, and a peripheral route, which is affective and non-critical, implying less cognitive elaboration (Petty and Cacioppo, 1986).

When elaboration likelihood is high, people will prefer central routes of persuasion (Petty and Cacioppo, 1984b), meaning that they will evaluate the positive and negative arguments with some care. However, when elaboration likelihood is low, people will prefer peripheral routes. These are characterized by cues external to the actual message, such as the external features of the transmitter or the quantity of arguments instead of their quality. LaMarre et al. (2014) observed that messages based on humor tend to decrease the recipients’ motivation to process the arguments underlying such messages, making it more likely for them to adopt the attitudes implicit in the message (Nabi et al., 2007), since exposure to humor implies a reduced willingness to argue against it (Baumgartner and Morris, 2008).

The elaboration likelihood model also posits that message repetition has an effect on persuasion, explained by a two-stage process (Cacioppo and Petty, 1979). When someone is exposed to a message, the repeated presentation of it can enhance the ability to process arguments. However, this process can also lead to a second stage in which repetition can produce tedium or reactance, and therefore decreased message acceptance by, for example, acting as a negative affective cue.

It is particularly interesting to consider the elaboration likelihood model when talking about the possible effects of being exposed to political disparagement humor and its duration because attitudes formed or changed by the peripheral route are less persistent (Petty et al., 2005). Also, as moderate repetition can have positive effects on persuasion, it can be hypothesized that a constant exposure to political disparagement humor will have effects on trust in politicians and that these effects will not be as short-lived as the ones caused by a one-time exposure.

Finally, evidence supports the idea that humor is processed via the peripheral rather than the central route (Zhang, 1996; Young, 2004; Baumgartner and Morris, 2008). Zhang (1996) found that humor (in the form of humorous advertisement) was more effective in the case of people that were low in need of cognition (i.e., people who are not predisposed to scrutinize and evaluate messages) and less effective in the case of people high in need of cognition. In the case of political humor, it has been observed that when people are presented with a humorous message which criticizes a political party, they tend to challenge less than if the message was presented seriously (Young, 2004).

## Political Disparagement Humor and Its Effects on Attitudes

According to Paletz (1990) humor directed against authority can be subverting, involving disparagement of political figures or ideologies, and can shape the attitudes of those who are exposed to this type of humor (Zenner, 1970; La Fave and Mannell, 1976). If disparagement humor makes negative stereotypes more accessible, the same stereotypes can take a person to have specific perceptions about targeted groups (Olson et al., 1999).

Effects of political disparagement humor on attitudes of those who are exposed to it have been a subject of a range of empirical studies (e.g., Hobden and Olson, 1994; Olson et al., 1999; Moy et al., 2006; Baumgartner et al., 2012; Baumgartner, 2013; Becker, 2014; Becker and Haller, 2014), though this research has not proved completely conclusive. This means that while some studies have not found any effects of the exposure to disparagement humor on attitudes (Olson et al., 1999), others have done so. For example, Baumgartner et al. (2012) found evidence suggesting that the impersonation of Sarah Palin by Tina Fey did achieve changes in attitudes toward her candidacy as Vice President (people who saw the spoof had a higher probability of disapproving her choice). Similarly, Hobden and Olson (1994) observed that after reading disparaging jokes about lawyers, people expressed more negative evaluations about them, which could lead to dissonance and therefore changes in attitudes (Olson et al., 1999). To summarize, though the existing research is not completely conclusive, most of the literature tends to acknowledge the effects of humor, including disparagement humor, on attitudes.

## Aims and Hypotheses

Two things can be concluded from the above review of the literature: firstly, political disparagement humor can have an effect on trust in politicians, as most of the previous research shows; secondly, this effect will be short-lived since humor probably implies less cognitive elaboration which leads to less persistent changes on attitudes, something that has been specifically addressed by other researchers such as Baumgartner and Morris (2008). However, moderate repetition of a message can help in reinforcing and changing attitudes. Thus, the following hypotheses guided this research:

• One-off exposure to political humor will have a negative effect on trust in politicians, but this effect will be short-lived (with the impact wearing off after a period of 1 week).
• Being exposed to political humor (through cartoons) on a daily basis will have a negative effect on trust in politicians, and this effect will not decrease after 1 week.

## Study 1

The first study sought to determine the effect of political stand-up comedy on people’s trust in politicians and whether the effect wears off over time. With this in mind, an experimental pretest–posttest control group design was created, with a first experimental group which was exposed to a video containing political disparagement humor, a second control group that was exposed to a video which showed instances of disparaging humor against regular, non-political citizens, and a third one exposed to a non-humorous political video. Trust in politicians was assessed in all the three groups at the baseline, immediately after the experimental manipulation, and once again a week later. As an incentive to take part in the experiment all participants had an equal chance to win a $50 gift voucher.

### Method and Procedure

#### Procedure

The questionnaire was programmed on 25 computers in a university laboratory. During a 2-week period, laboratory sessions were held at the university campus. The experiment was explained to the participants in the campus in broad terms by two research assistants. After that, those who accepted to take part in the experiment were taken to the laboratory and were asked to read and sign an informed consent.

A baseline questionnaire which included the dependent variable, trust in politicians, along with disposition toward politicians, political affiliation, and assessment of sex and age was presented to the volunteers (Time 1). After having completed the baseline questionnaire, they were automatically and randomly assigned to one of the three conditions and were exposed to the respective stimuli.

Once they had watched each video, a second questionnaire was presented to them to be filled out containing the dependent variable, an assessment of cognitive elaboration, and of funniness and aversiveness (Time 2). Finally, 1 week later, they were sent an electronic link with a third questionnaire containing the dependent variable (Time 3).

#### Sample

We used the G^∗^Power software (Faul et al., 2009) to determine the minimum sample size required for obtaining a significant medium effect size (*f* = 0.25), given α = 0.05, and a statistical power of 0.80, assuming no correlation between measures. With this analysis, we estimated a minimum sample size of 69 individuals. One hundred and fifty-eight undergraduate students participated in Study 1, and were randomly assigned to each of the three groups. Sixty-two participants were dismissed from analyses because they either (a) failed in watching the video – which was inferred from the time they took in completing the study – or (b) did not follow the instructions appropriately (for example, used their phones, talked with other participants during the experiment, or opened web pages on the computer). Attrition followed a random pattern, given that no significant differences were found between those participants who were considered in the final sample and participants who were not, either by sociodemographic characteristics, such as age, *F*(1,156) = 0.121, *p* > 0.05, η^2^ = 0.001, and sex, χ^2^(1) = 3.599, *p* > 0.05, or baseline trust, *F*(1,154) = 2.564, *p* > 0.05, η^2^ = 0.016. Fifty-one percent of the participants were male, and the mean of the age was 20.96 (*SD* = 2.15). Descriptive statistics for the sample are presented in **Table 1**.

**Table 1:** Sex and age descriptive statistics for the three groups.

Group	*n*	Sex		Age (*SD*)
			
		Men	Women	
Political disparagement humor	31	51.60%	48.40%	20.90 (2.06)
Disparagement humor against non-politicians	34	46.90%	53.10%	20.75 (2.00)
Non-humorous political video	31	54.80%	45.20%	21.23 (2.40)


#### Stimuli

Three videos were used. For the videos containing humor (i.e., experimental group and the control group exposed to the disparagement humor against the non-politicians video) two edited stand-up comedy routines were used. Both were by the same comedian (Edo Caroe, a popular Chilean stand-up comedian and magician), which aired in 2015 and 2016 and presented on the Festival of Viña del Mar and the Festival del Huaso de Olmué, both Chilean festivals with live transmission to Latin America. The presentations were edited to have similar duration (12 min and 32 s for the experimental video and 13 min and 2 s for control video containing humor) and to ensure that their content would be in line with the aims of the study.

To assess the validity of the videos that we used, we asked four evaluators (university students) to rate four statements about the experimental video and three about the control video. The statements are displayed in **Table 2**.

**Table 2:** Statements presented to assess stimuli validity.

Political humor (experimental)	Non-political video (control)
It is funny	It is funny
It is political humor	It is political humor
There is disparagement	There is disparagement
It does not specially attack politicians of a political party, but instead criticizes transversally	–


To do this, the raters had to answer “yes” or “no” to each statement. In every case, each rater agreed on the same answer. In the case of the experimental video, the four raters answered “yes” to all the statements. In the case of the control video, the four raters answered “yes” to the statements “*it is funny*” and “*there is disparagement*” and “no” to the statement “*it is political humor.*”

Parts of the transcription of the video which used political disparagement humor are below:

For example Senator Pizarro. When his region most needed him, he traveled to England in order to attend a rugby match. Rugby has always been a gentleman’s sport, what was he doing there?! If he wanted to see dirty people, he could have gone to La Moneda!
*Politicians in Chile are dumb. They were bought by big enterprises, write useless laws. For example, Jaime Orpis received bribes from Corpesca. Money, real money! Bribes in Chile are strange: I always thought that bribes involved two men with suits, sunglasses in a dark alley leaving a suitcase, or at a restaurant, passing a suitcase under the table. It is different in Chile. Here politicians give you a receipt. Let’s be corrupt but keep things in order. Jaime Orpis is so stupid that he even wrote on the receipt* “*Bribe May 2015*.”
*Every time our politicians are on TV, no one thinks “oh, great, our politicians on TV, let’s see what the new social advance is.” No, it’s “what did they do now?”. That happens to me every time I see Gustavo Hasbún. Every time. Have you ever seen someone more stupid than Hasbún? He’s so stupid that idiots refer to themselves as* “*Hasbún*.”
*Let’s take a look at the example of Dávalos* (Michele Bachelet’s son). *He got rich using his position and, not happy with that, he erased everything, all the evidence that was on his computer. You might even say he was something like Pinochet. He tried to eliminate the PC* (Note: in Spanish, PC can also mean “partido comunista” or “communist party”).
*Let’s be clear, the president has had bad luck. She has been a lousy manager but she has had bad luck. The other day I saw a black cat that was very scared because it met Bachelet. She never knows anything! One day she met Daddy Yankee and he told her* “*You know*” (Note: his catchphrase) *and the lady didn’t know!*

Some parts of the transcription of the video using disparagement humor against non-politicians are below:

You can’t imagine how nervous I am of being here. I wasn’t this nervous since my wife gave birth. I hope Birth will be able to forgive us when she grows up.
My mother always said that when you go somewhere where no one knows you, you must introduce yourself, so well, my name is Edo Caroe, I’m a comedian, I come from Temuco. Somebody from Temuco here? A horrible city, that’s why I left. No, no, sorry. Just kidding. I’m proud of Temuco, I have always been.
I decided to become a comedian just to see my father smile. I then found out that I should have been a doctor, since he has a horrible facial paralysis.
My father just learned how to use WhatsApp and he spends the whole day sending nude pictures of naked women to me. He’s a forensic expert.
I’ve always liked humor, maybe because it has always been difficult for me to be still and not move. My mother always remembers how I kicked her belly. Especially when she was pregnant with my sister.
I love my daughter. She is older now, she lost her first tooth yesterday. I apologized and promised I wouldn’t drink again. Last night she went to our room exactly when my wife was having an orgasm. It was a very uncomfortable moment for me and my friends, but it was a good opportunity to teach her about teamwork.
*I’ve always wanted to come to this city. My family was very happy for me, my grandmother who has diabetes was jumping on one leg. The other one had been amputated. But I was not sure if I should come. I thought it would be difficult, more difficult than playing Scrabble with a dyslexic kid. I decided to come because I like risks. I like risks so much, that if Johnny Herrera* (Note: a football player involved in a car accident) *offers to give me a lift, I say yes. Really. I once bungee jumped from Lucho Jara’s ego* (Note: a famous TV host). *And most people don’t understand those that like taking risks. Loving risk is going to “Who wants to be a millionaire” and use “Ask a Friend” to call Arturo Longton.*

The second control group was exposed to a non-humorous political video. This was a video blog by the Chilean journalist Tomás Mosciatti. To assess its validity the same questions as the ones used with the humorous videos were used with the same four raters. They all agreed that it was not funny, that it was disparaging, that it had political content, and that it attacked targets across the political spectrum.

#### Instruments

##### Trust in politicians

A modified version of the Yamagishi and Yamagishi’s General Trust Scale was used (Yamagishi and Yamagishi, 1994) replacing “people” by “politicians.” It was assessed by means of a 100-point scale that ranged from total disagreement to total agreement and covered the following statements: “*Most politicians are essentially honest*,” “*Most politicians are essentially good and kind*,” “*Most politicians are trustworthy*,” and “*Most politicians will respond kindly when they are trusted by others.*” Scale reliability was high for the baseline questionnaire (α = 0.76), the second questionnaire (α = 0.79), and the third questionnaire (α = 0.82).

##### Disposition toward politicians

It was assessed with the item “*How much would you say you like politicians?*” with responses ranging from 1 (*Do not like*) to 100 (*Like very much*).

##### Political affiliation

Participants were asked about their political ideology according to a left-right political spectrum, for which possible responses were “*Left wing*,” “*Center-Left wing*,” “*Center*,” “*Center Right Wing*,” “*Right Wing*,” or “*None of the above.*”

##### Funniness

It was assessed with one item that ranged from 1 (*not funny*) to 100 (*very funny*).

##### Aversiveness

It was assessed with one item that ranged from 1 (*no aversiveness*) to 100 (*high aversiveness*).

##### Cognitive elaboration

It was assessed using a modified version of the scale created by Igartua (2010), considering a 100-point scale that varied between total disagreement and total agreement to the following statements: “*I have reflected on the topic it dealt with*,” “*I have thought about the situation and the motivations of the characters*,” “*I have tried to see how the plot was related to other topics that interest me*,” and “*I have wanted to draw some conclusions about the topic addressed here*.” Scale reliability was high (α = 0.81).

### Results and Discussion

#### Manipulation Checks

First, the experimental manipulation was checked. A univariate ANOVA revealed significant differences in funniness, *F*(2,91) = 93.261, *p* < 0.001, η^2^ = 0.672. The Tukey *post hoc* test showed that the control group exposed to the non-humorous political video (*M* = 14.00, *SD* = 19.689) was different from both the experimental group (*M* = 80.61, *SD* = 24.52) and the control group exposed to the disparagement humor against non-politicians video (*M* = 75.28, *SD* = 19.64), *p* < 0.001 in both cases. In addition, there were no significant differences in aversiveness among the groups, *F*(2,91) = 2.559, *p* > 0.05, η^2^ = 0.053. These results suggest that the manipulation through exposure to video was successful.

Means and standard deviations for the three groups regarding trust at times 1, 2, and 3, funniness, aversiveness, and cognitive elaboration can be found in **Table 3**.

**Table 3:** Means and standard deviations for trust times 1, 2, and 3, funniness, aversiveness, and cognitive elaboration.

		*M*	*SD*
Trust time 1	Political disparagement humor	30.23	13.72
	Disparagement humor against non-politicians	28.76	16.18
	Non-humorous political video	30.20	15.27
Trust time 2	Political disparagement humor	23.56	9.79
	Disparagement humor against non-politicians	30.78	15.37
	Non-humorous political video	23.25	17.38
Trust time 3	Political disparagement humor	29.06	13.03
	Disparagement humor against non-politicians	28.82	13.10
	Non-humorous political video	28.57	16.45
Funninness	Political disparagement humor	80.61	24.51
	Disparagement humor against non-politicians	75.00	19.73
	Non-humorous political video	14.00	19.69
Aversiveness	Political disparagement humor	30.00	24.80
	Disparagement humor against non-politicians	32.12	23.69
	Non-humorous political video	20.58	25.71
Cognitive Elaboration	Political disparagement humor	68.69	15.64
	Disparagement humor against non-politicians	46.10	19.34
	Non-humorous political video	63.48	21.98


#### Main Analyses

We controlled for disposition and political affiliation by using randomized groups. In this case, no differences between the groups regarding both variables were found.

The first hypothesis refers to the cognitive elaboration that each stimulus implied, so as to observe if less elaboration was being used in the case of humorous stimuli. A univariate ANOVA showed significant differences regarding this variable, *F*(2,91) = 12.875, *p* < 0.000, η^2^ = 0.223. The main differences were observed contrasting the control group exposed to the video presenting disparagement humor against non-politicians (*M* = 45.30, *SD* = 19.58) to both the experimental group (*M* = 68.69, *SD* = 15.64) and the control group exposed to the non-humorous political video (*M* = 63.48, *SD* = 21.98), *p* < 0.001 in both cases.

The second hypothesis, and the core of Study 1, refers to the effects of political humor on political trust. A 3 (condition) by 3 (time) ANOVA with repeated measures was performed. The results showed a significant main effect of the measures of political trust, *F*(2,182) = 6.344, *p* < 0.01, $\eta_{p}^{2}$ = 0.065, but not of the group, *F*(1,91) = 0.405, *p* > 0.05, $\eta_{p}^{2}$ = 0.009. Nevertheless, and more importantly, the interaction between both factors was significant, *F*(4,182) = 3.949, *p* < 0.01, $\eta_{p}^{2}$ = 0.080. It is important to note that for the effects of the measures of political trust and the interaction between the factors, the observed power was high (0.896 and 0.900, respectively). When controlled by either funniness or aversiveness, a similar pattern of results was found, obtaining in both cases the same significant interaction term, *F*(4,180) = 3.384, *p* < 0.01, $\eta_{p}^{2}$ = 0.070, and *F*(4,180) = 3.528, *p* < 0.01, $\eta_{p}^{2}$ = 0.073, respectively.

Given these results, we contrasted the effects of group on political trust, for each measure separately. For the baseline, we found no significant differences by group, *F*(2,91) = 0.007, *p* > 0.05, η^2^ = 0.000. In the first post-measure, we observed significant differences, *F*(2,91) = 3.241, *p* < 0.05, η^2^ = 0.066. The *post hoc* analysis revealed that the control group exposed to the video presenting disparagement humor against non-politicians (*M* = 31.50, *SD* = 15.56) was marginally different from both the experimental (*M* = 23.56, *SD* = 9.79), *p* < 0.1, and the control groups exposed to the non-humorous political video (*M* = 23.25, *SD* = 17.38), *p* < 0.1. Finally, in the second post-measure, there were no significant differences between the groups, *F*(2,91) = 1.815, *p* > 0.05, η^2^ = 0.000.

**FIGURE 1:**
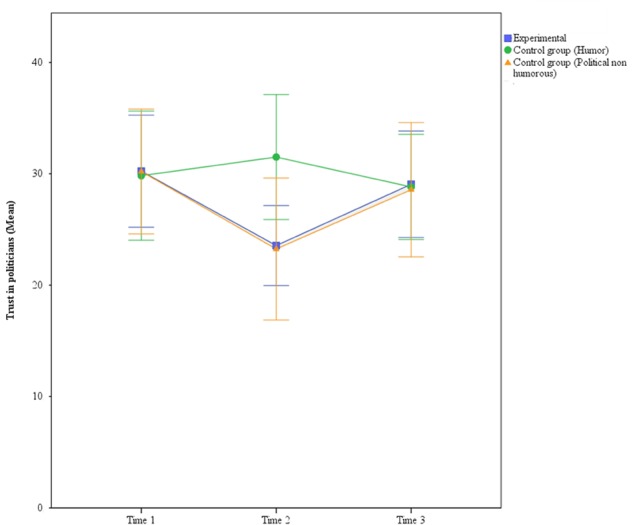
Trust in politicians at times 1, 2, and 3 (Study 1).

In sum, the overall pattern of results suggests that both groups exposed to political content declined in political trust immediately after viewing the video, but returned to the baseline levels 1 week later, as it is shown in **Figure 1** (each error bar is constructed using a 95% confidence interval of the mean).

It must be considered that these results are independent of either the perceived funniness or the perceived aversiveness, as was shown earlier. A supplementary analysis showed that funniness was not significantly related to trust in the baseline, the second measure, and the last measure for the experimental group, with similar results for those participants assigned to control group 1 and those assigned to control group 2. The same pattern of results was obtained analyzing the relationship between aversiveness and the three measures of trust for those assigned to the experimental group, for those assigned to the first control group, and those assigned to the second control. In addition, there was no relationship between funniness and elaboration, neither for the experimental group nor for both the control group exposed to disparagement humor and the control group exposed to the non-humorous political video. This can be observed in **Table 4**.

**Table 4:** Correlations between considered variables.

		Trust time 1	Trust time 2	Trust time 3	Funninness	Aversiveness	Political affiliation	Disposition toward politicians
Political disparagement	Trust time 2	0.57**	1.00					
humor	Trust time 3	0.69**	0.60**	1.00				
	Funninness	-0.17	-0.20	-0.21	1.00			
	Aversiveness	-0.35	0.17	-0.01	-0.36*	1.00		
	Political affiliation	-0.03	0.02	-0.31*	-0.15	0.06	1.00	
	Disposition toward politicians	0.42**	0.69**	0.43**	-0.15	0.04	0.02	1.00
	Cognitive elaboration	-0.28	-0.45**	-0.38*	0.16	-0.35*	-0.14	-0.25
Disparagement humor	Trust time 2	0.80**	1					
against non-politicians	Trust time 3	0.75**	0.82**	1				
	Funninness	0.15	0.11	0.05	1			
	Aversiveness	-0.10	-0.07	0.03	-0.20	1		
	Political affiliation	-0.07	0.07	0.09	-0.03	-0.1	1	
	Disposition toward politicians	0.67**	0.70**	0.64**	0.07	-0.11	0.02	1
	Cognitive elaboration	0.15	-0.01	0.10	0.31*	0.13	-0.20	-0.08
Non-humorous political	Trust time 2	0.70**	1.00					
video	Trust time 3	0.72**	0.74**	1.00				
	Funninness	0.13	0.13	0.11	1.00			
	Aversiveness	-0.19	0.04	-0.09	0.09	1.00		
	Political affiliation	0.06	0.19	0.18	-0.13	0.27	1.00	
	Disposition toward politicians	0.60**	0.51**	0.44**	-0.10	-0.21	0.19	1.00
	Cognitive elaboration	0.06	-0.06	0.02	0.09	-0.06	-0.27	-0.15


*^∗^*p* < 0.05, ^∗∗^*p* < 0.01.*

#### Discussion

Results from study 1 show two key elements of this research. On the one hand, it was observed that the effect of political disparagement humor on individuals tends to be similar to the effect of political information that is non-humorous. This can be due to the fact that political humor implies more cognitive elaboration than non-political humor, even at the same level of political non-humorous information. On the other hand, it was also observed that the effects in both cases did not last long, being, as hypothesized, short-lived.

One topic is still open regarding whether constant presentation of a stimulus for a long period of time implies long-term effects. With the intention of addressing this, a second study was designed.

## Study 2

The second experiment aimed to find evidence on the way that being exposed to political humor (in the form of cartoons) on a daily basis might impact trust of the individuals in politicians. For this purpose, a pretest–posttest control group design was used. Participants were university students at the university campus. They first received the baseline questionnaire which contained assessments of trust in politicians, political affiliation, disposition toward politicians, exposure to political humor, exposure to political information, sex, age, and WhatsApp number. After this, participants were randomly assigned either to an experimental or to one of two control groups, which received different stimuli via WhatsApp twice a day for 1 week. The experimental group received political cartoons; the first control group received non-political cartoons; and the second control group received newspaper headlines regarding political topics (such as conflicts of interests). Trust in politicians and attention paid to the stimuli among the three groups were assessed again after 1 week and a third time after 2 weeks via WhatsApp. As in study 1, as an incentive to take part in the experience, all participants had an equal chance to win three $50 gift vouchers.

### Method and Procedure

#### Procedure

A research assistant contacted the participants on the university campus and explained the general aim of the study and the procedure. They were given an informed consent document that explained the study in detail. After agreeing to participate in the study, the participants were given a questionnaire with baseline questions (Time 1) containing the dependent variables (trust in politicians), political affiliation, disposition toward politicians, exposure to political humor, exposure to political information, sex, age, and a WhatsApp number. Starting the next day and for 7 days, the stimuli were sent via WhatsApp to the participants who were randomly assigned to the experimental group (political cartoons), the first control group (non-political cartoons), and the second control group (newspaper headlines regarding political topics). After that week, the same questions assessing trust in politicians were sent to the experimental and control groups (Time 2). Finally, 1 week later, the three groups were sent the same questions (Time 3).

#### Sample

We used the GPower software (Faul et al., 2009) to determine the minimum sample size required, considering the effect size obtained in study 1 (*f* = 0.29), given α = 0.05, and a statistical power of 0.80, assuming no correlation between measures. With this analysis, we estimated a minimum sample size of 78 individuals. Three hundred and forty-seven students participated in the baseline (59.1% women, *M*_age_ = 20.90, *SD* = 1.73). One hundred and ninety-seven of them sent their responses back after 1 week (55.8% women, *M*_age_ = 20.83, *SD* = 1.69). Finally, 146 sent their responses back 1 week after that (50.7%women, *M*_age_ = 20.81, *SD* = 1.67). It can be established that there are no differences between those who were part of the final sample and those who were not, since attrition followed a random pattern. No significant differences were found between the two groups regarding age, *F*(1,345) = 0.738, *p* > 0.05, η^2^ = 0.002, sex, χ^2^(1) = 2.573, *p* > 0.05, baseline trust, *F*(1,343) = 2.762, *p* > 0.05, η^2^ = 0.008 and disposition toward politicians, *F*(1,345) = 0.997, *p* > 0.05, η^2^ = 0.003. Descriptive statistics for the sample are presented in **Table 5**.

**Table 5:** Sex and age descriptive statistics for the three groups Study 2.

Group	*n*	Sex		Age (*SD*)
			
		Men	Women	
Political cartoons	54	57.4%	42.6%	20.85 (1.83)
Non-political cartoons	43	46.5%	53.5%	20.84 (1.59)
Newspaper headlines	49	42.9%	57.1%	20.73 (1.60)


#### Stimuli

**FIGURE 2:**
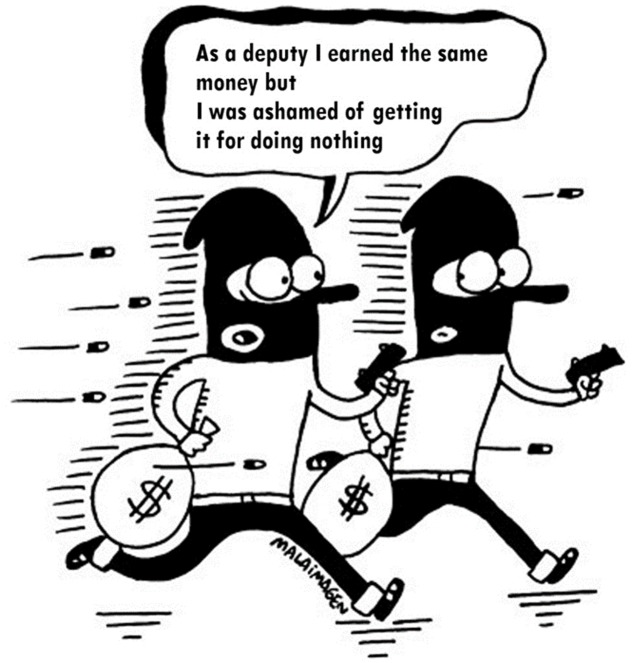
Example of cartoon used for study 2.

We used 14 political cartoons selected from image databases that implied criticism toward politicians in general, with no party-political bias. None referred to a politician or political figure identified by name or appearance. Two university students rated the 14 cartoons with complete agreement, evaluating disparagement (“*There is disparagement*” with response options being “yes” and “no”), if they were political (“*It is political humor*” with response options being “yes” and “no”), and if they were transversal (“*It does not specially attack politicians of a political party, but instead criticizes transversally*” with response options being “yes” and “no”). An example of a cartoon by the Chilean cartoonist Malaimagen is displayed in **Figure 2**. In the case of the 14 non-political cartoons and the 14 newspaper headlines there was also agreement.

#### Instruments

##### Trust in politicians

We used the same adaptation used in study 1. It presented adequate internal consistency considering Cronbach’s Alpha at times 1, 2, and 3 (0.83, 0.84 and 0.89, respectively).

##### Attention

We asked the participants to rate how much attention they pay to the stimuli after the 1st week (1 = No attention; 100 = Total attention).

##### Disposition toward politicians

It was assessed with the item “*How much would you say you like politicians?*” with responses ranging from 1 (*Do not like*) to 100 (*Like very much*).

##### Political affiliation

Participants were asked about their political ideology according to a left–right political spectrum, for which possible responses were “*Left wing*,” “*Center-Left wing*,” “*Center*,” “*Center Right Wing*,” “*Right Wing*,” or “*None of the above.*”

##### Exposure to political humor

We used the item “*How often do you watch shows or read websites or newspapers that make fun of politicians?*” (1: Almost never; 10: Always).

##### Exposure to political information

We used the item “*How often do you watch shows or read websites or newspapers that refer to politics?*” (1: Almost never; 10: Always).

Funniness and aversiveness were not assessed. This decision was made due to the characteristics of the design, and given that it would have involved asking participants to rate the stimuli twice a day for 7 days, which could have led to higher attrition (57.9%). Considering that, we decided to assess trust and attention after the exposure to the stimuli, since funniness and aversion had already been rated by two raters. This is discussed in the limitations sections.

### Results

**FIGURE 3:**
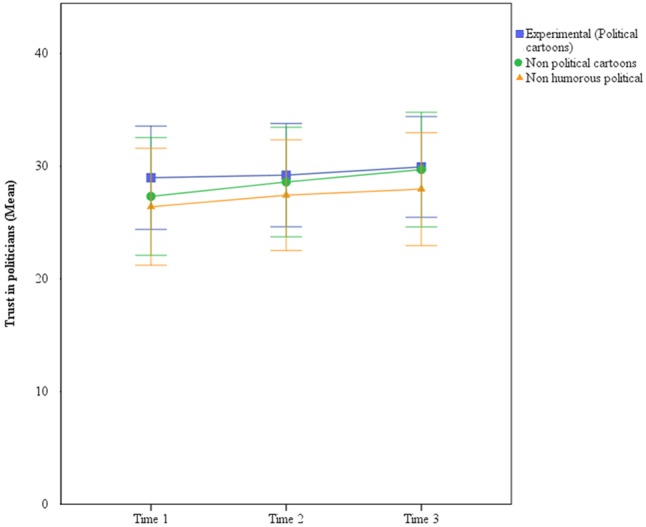
Trust in politicians at times 1, 2, and 3 (Study 2).

The three groups were not different regarding sex, χ^2^(2,146) = 2.368, *p* > 0.05, age, *F*(2,45) = 1.287, *p* > 0.05, η^2^ = 0.057, political affiliation, *F*(2,143) = 0.071, *p* > 0.05, η^2^ = 0.001, or disposition toward politicians, *F*(2,143) = 1.176, *p* > 0.05, η^2^ = 0.016. With this in mind, the decision was made to repeat the analysis of Study 1. In this case, a 3 (condition)-by-3 (time) ANOVA with repeated measures in the last factor was performed. The results showed no significant effect of either condition, *F*(2,143) = 0.226, *p* > 0.05, $\eta_{p}^{2}$ = 0.003, time, *F*(2,286) = 2.078, *p* > 0.05, $\eta_{p}^{2}$ = 0.014, or the interaction term, *F*(4,286) = 0.153, *p* > 0.05, $\eta_{p}^{2}$ = 0.002. The observed power for the three terms was low (0.085, 0.426, and 0.082, respectively). However, this should not be considered as a reason to discard this results since – as it will be discussed in the conclusions section – non-significant results can correspond to low observed power (Hoenig and Heisey, 2001). Means and confidence intervals for each condition in times 1, 2, and 3 can be observed in **Figure 3** (each error bar is constructed using a 95% confidence interval of the mean). Means and standard deviations for each group at times 1, 2, and 3 can be found in **Table 6**.

**Table 6:** Means and standard deviations for trust times 1, 2, and 3, attention, exposure to political humor and exposure to political information (Study 2).

		*M*	*SD*
Trust time 1	Political cartoons	28.97	16.79


	Non-political cartoons	27.32	16.98


	Newspaper headlines	26.40	18.06


Trust time 2	Political cartoons	29.20	16.79


	Non-political cartoons	28.59	15.79


	Newspaper headlines	27.42	17.12


Trust time 3	Political cartoons	29.93	16.39


	Non-political cartoons	29.70	16.53


	Newspaper headlines	27.96	17.47


Attention	Political cartoons	81.73	31.08
	Non-political cartoons	80.07	23.09
	Newspaper headlines	73.92	27.44
Exposure to political humor	Political cartoons	5.65	2.69
	Non-political cartoons	4.38	2.54
	Newspaper headlines	4.78	2.26
Exposure to political information	Political cartoons	5.13	1.98
	Non-political cartoons	5.21	2.48
	Newspaper headlines	5.03	2.46


We performed three supplementary analyses including attention, exposure to political information, and exposure to political humor as covariates in separated models, but we obtained similar results. Specifically, when attention was included, we observed non-significant effects of either condition, *F*(2,101) = 0.523, *p* > 0.05, $\eta_{p}^{2}$ = 0.010, time, *F*(2,202) = 2.666, *p* > 0.05, $\eta_{p}^{2}$ = 0.026, attention, *F*(1,101) = 0.002, *p* > 0.05, $\eta_{p}^{2}$ = 0.000, the interaction term between time and condition, *F*(4,202) = 0.128, *p* > 0.05, $\eta_{p}^{2}$ = 0.003, and the interaction term between time and attention, *F*(2,202) = 1.861, *p* > 0.05, $\eta_{p}^{2}$ = 0.018. The observed power for the terms was 0.134, 0.525, 0.050, 0.076, and 0.385, respectively. When exposure to political information was included, there were no significant effects of either condition, *F*(2,101) = 0.524, *p* > 0.05, $\eta_{p}^{2}$ = 0.010, time, *F*(2,202) = 2.885, *p* > 0.05, $\eta_{p}^{2}$ = 0.028, exposure to political information, *F*(1,101) = 0.117, *p* > 0.05, $\eta_{p}^{2}$ = 0.001, the interaction term between time and condition, *F*(4,202) = 0.157, *p* > 0.05, $\eta_{p}^{2}$ = 0.003, and the interaction term between time and exposure to political information, *F*(2,202) = 1.928, *p* > 0.05, $\eta_{p}^{2}$ = 0.019. The observed power for the terms was 0.134, 0.560, 0.063, 0.083, and 0.397, respectively. Finally, when we included exposure to political humor, there were no significant effects of either condition, *F*(2,101) = 0.389, *p* > 0.05, $\eta_{p}^{2}$ = 0.019, time, *F*(2,202) = 0.595, *p* > 0.05, $\eta_{p}^{2}$ = 0.006, exposure to political humor, *F*(1,101) = 2.980, *p* > 0.05, $\eta_{p}^{2}$ = 0.029, the interaction term between time and condition, *F*(4,202) = 0.250, *p* > 0.05, $\eta_{p}^{2}$ = 0.005, and the interaction term between time and exposure to political humor, *F*(2,202) = 0.987, *p* > 0.05, $\eta_{p}^{2}$ = 0.010. The observed power for the terms was 0.211, 0.148, 0.401, 0.104, and 0.220, respectively.

## Conclusion

In general terms, the obtained results point in the expected direction in most cases, but there are at least two elements that are worth considering. According to what was observed in study 1, political disparagement humor has an effect on trust in politicians. However, trust in politicians returns to the same level as the control groups in a second post-exposure measurement. It seems to be that humor can affect attitudes temporarily, but does not change them permanently. These results are in accordance with earlier findings (Weinberger and Gulas, 1992; Olson et al., 1999). Although the result in the present study was expected, the explanatory pathways of the phenomenon are not clear.

On the one hand, it was hypothesized that the reason for this short-lived effect would be that humor is processed through the peripheral route, understood as less cognitive elaboration. Our results do not support this, since political disparagement humor and non-humorous disparagement political information did not show differences between them regarding the degree of cognitive elaboration. However, both of them showed higher cognitive elaboration than the non-political disparagement humor group. That is to say, humor did imply less cognitive elaboration, but disparagement political humor did not.

Therefore, it is not possible in this case to positively state that the limited durability of the effects of political disparagement humor on attitudes toward politicians can be explained because humor communicates through a peripheral route, decreasing the motivation to counter-argue against the message (Baumgartner and Morris, 2008).

On the other hand, the behavior of two of the groups of study 1 was almost identical. Both the experimental group and the control group exposed to the video with disparagement non-humorous political content showed decreases in the first post-exposure evaluation, being different from their previous measurements and the control group exposed to non-political disparagement humor.

The conclusion to these two aspects seems to be the same: it looks as if political humor is not different from other ways of communicating political content regarding its effects on trust in politicians. This, considering that all the groups were comparable in relation to political affiliation and disposition toward the politicians, would imply that although there could be a positive or a negative disposition toward politicians, disparagement political content has an effect in any form in which it is presented.

It is also necessary to refer to the results of study 2. In this case, there were no effects of political humor on trust in politicians, or any of the relationships that were observed in study 1. Two ideas may help explain these results.

The first one refers to the degree of control that experiments of these characteristics can have. This was not an experiment run in a laboratory, which makes it difficult to assure that participants pay proper attention to the stimuli, for example, even when in this case we did ask participants to rate how much attention they pay to the stimuli.

The second idea refers to theoretical implications of the results of study 2. The type of stimulus used in study 1 was audiovisual, whereas in study 2, only graphic stimuli were used. There may be something in the content and the form of a more complex stimulus that arouses more attention and could therefore generate effects on trust.

There is also the topic of interest in the exposure to political material. It could be thought that forcing a person to consume material daily without any particular motivation would have no effect. In other words, it could be expected that those people who are more interested in consuming political humor could have their attitudes affected (or changed) for a longer period because they would constantly be in contact with stimuli of this kind. As Baumgartner (2013) suggests, it is possible to think that those who are more interested in politics and politicians are not only going to be more interested in consuming information about it, but also would be more interested in consuming political humor and, within it, political disparagement humor recurrently. However, our results do not show an effect of any of these variables on trust in politicians.

This research has limitations. For example, we have considered a measure of cognitive elaboration, but there are other ways of assessing this variable, like thought listing tasks, that could help as a useful complement. Finding other ways of exposing participants to political disparagement humor for longer periods of time would also be useful and could help improving the design of similar experiments.

The validity of the stimuli is essential in an experiment. In this case, we tried to assess such validity by asking two students to rate different elements of the videos and images used in both studies. However, the rating involved dichotomous answers (“yes/no”) which could imply not being able to have an idea of the magnitude of possible differences, even though there was complete agreement in every evaluation and the manipulation checks suggest that the stimuli worked properly. It is then possible that the final results could be caused by differences in this magnitude and not the exposition to different stimuli. However, the manipulation checks showed that all the evaluations of the stimuli were as expected and in an expected direction, which is an indicator of a good selection and that the observed effects were very probably caused by the independent variable.

Another evident limitation is not having assessed funniness and aversiveness in study 2. We were forced to make this decision because the design of our experiment would have involved asking participants to rate the stimuli twice a day for a week, which would, we consider, generate higher attrition. Aversiveness and funniness are two basic components of the response to humor, so not considering its impact on participants could involve two things: one, that what we supposed would be disparagement humor was not in fact disparagement (not eliciting more aversiveness than other stimuli) and two, that the stimuli would not be in fact considered funny. Both elements would have an impact on trust, considering our design and the aims of this study. In this case we still had an evaluation of the stimuli given that two raters evaluated them, but not having the participants rate both variables and not being able to control for them (as it was possible in study 1, with clearer results about the role of both variables in the relation between exposure and trust) is a limitation of study 2 that we had to accept.

Finally, a last possible limitation is the low observed statistical power of Study 2. The method used in both studies to determine sample sizes considering a power of 0.80 with GPower was good enough in study 1 but not in study 2. Nevertheless, we think our results are reliable, given that we exceed the minimum sample size when power was computed *a priori*. In addition to this, the *post hoc* procedure of power calculation has been criticized by different authors because it depends on the observed *p*-value and non-significant *p*-values might correspond to low observed powers (Goodman and Berlin, 1994; Hoenig and Heisey, 2001).

We have seen that the effect is short-lived, but when exactly does disparagement humor stop affecting trust in politicians? Which other variables could help by amplifying or weakening that effect? This research also showed that disparagement political humor was not cognitively processed as non-political humor, which presents an interesting line of research. We think that this research is a step forward, not only considering its results, but also considering the questions that arise from it.

## Ethics Statement

This study was carried out in accordance with the recommendations of the ethics committee of the University of Santiago with written informed consent from all subjects. All subjects gave written informed consent in accordance with the Declaration of Helsinki. The protocol was approved by the ethics committee of the University of Santiago.

## Author Contributions

All the authors listed have made a substantial, direct, and intellectual contribution to the work, and approved it for publication.

## Conflict of Interest Statement

The authors declare that the research was conducted in the absence of any commercial or financial relationships that could be construed as a potential conflict of interest.
